# Supplementary figures and images for: Where is my arm? Investigating the link between complex regional pain syndrome and poor localisation of the affected limb
Source: PeerJ. 2021 Aug 20;9:e11882. doi: 10.7717/peerj.11882 (PMC8381877; doi:10.7717/peerj.11882)

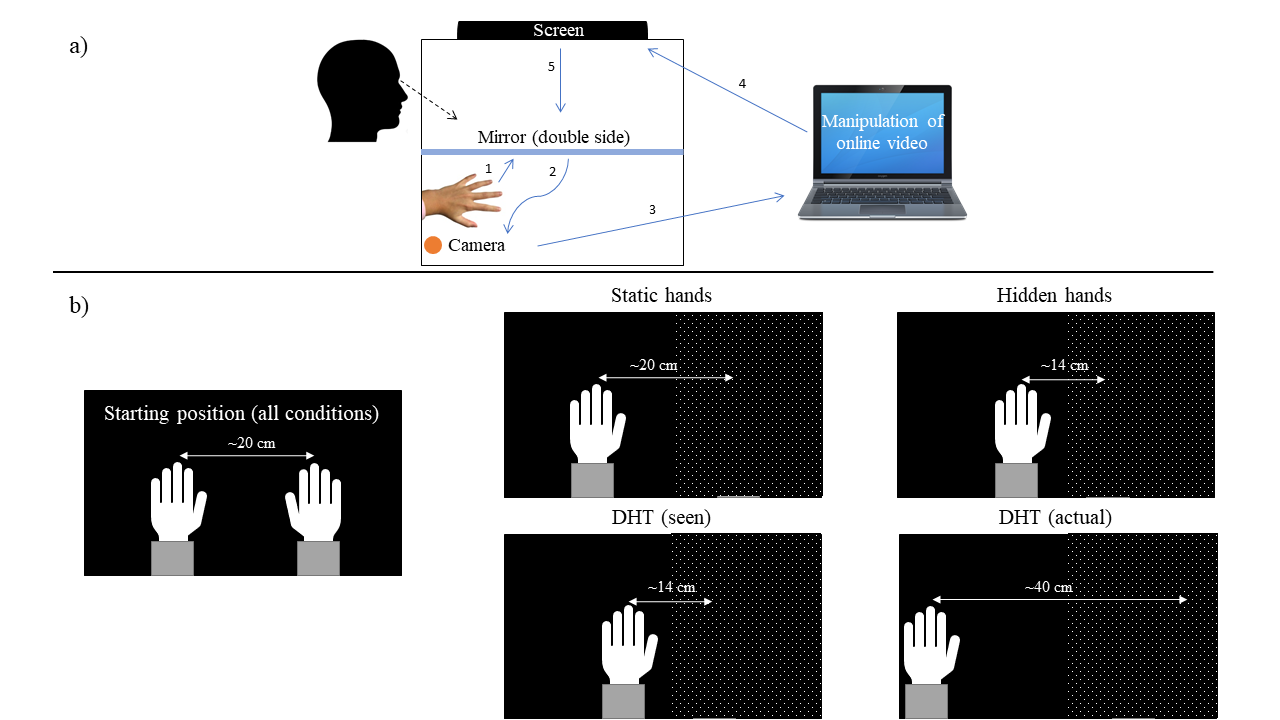

Supplement: Supplemental Information 5 [file peerj-09-11882-s005.png]

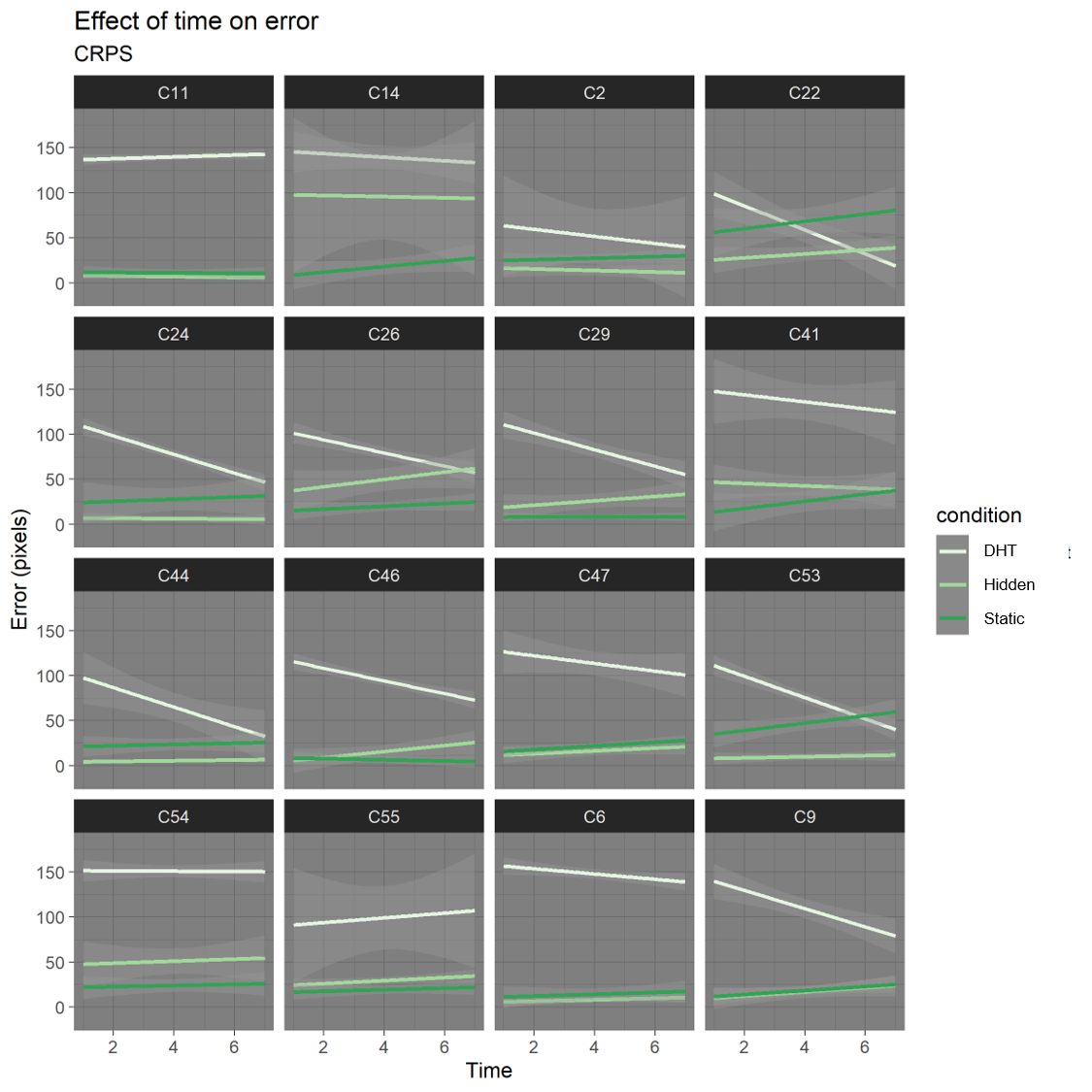

Supplement: Supplemental Information 6 [file peerj-09-11882-s006.png]
