# Supplementary material for: Where is my arm? Investigating the link between complex regional pain syndrome and poor localisation of the affected limb
Source: PeerJ. 2021 Aug 20;9:e11882. doi: 10.7717/peerj.11882 (PMC8381877; doi:10.7717/peerj.11882)
Supplement: Supplemental Information 7 [file peerj-09-11882-s007.docx]

**Table S1:**

**CRPS diagnostic criteria. Adapted from (Harden, Bruehl, Stanton-Hicks, & Wilson, 2007)**

| 1. | Continuing pain, which is disproportionate to any inciting event. | | |
| --- | --- | --- | --- |
| 2. | Symptoms (at least 1 in 3 of the 4 categories) | SENSORY | Hyperesthesia and/or allodynia. |
|  |  | VASOMOTOR | Temperature asymmetry and/or skin colour changes and/or skin colour asymmetry. |
|  |  | SUDOMOTOR/OEDEMA | Oedema and/or sweating changes and/or sweating asymmetry. |
|  |  | MOTOR/TROPHIC | Decreased range of motion and/or motor dysfunction (weakness, tremor, and dystonia) and/or trophic changes (hair, nail, and skin). |
| 3. | Signs at the time of evaluation (at least 1 in 2 or more of the 4 categories) | SENSORY | Hyperalgesia (to pinprick) and/or allodynia (to light touch and/or temperature sensation and/or deep somatic pressure and/or joint movement) |
|  |  | VASOMOTOR | Temperature asymmetry (>1°C) and/or skin colour changes and/or asymmetry |
|  |  | SUDOMOTOR/OEDEMA | Oedema and/or sweating changes and/or sweating asymmetry. |
|  |  | MOTOR/TROPHIC | Decreased range of motion and/or motor dysfunction (weakness, tremor, and dystonia) and/or trophic changes (hair, nail, and skin). |
| 4. | There is no other diagnosis that better explains the signs and symptoms | | |
