# Supplementary material for: Where is my arm? Investigating the link between complex regional pain syndrome and poor localisation of the affected limb
Source: PeerJ. 2021 Aug 20;9:e11882. doi: 10.7717/peerj.11882 (PMC8381877; doi:10.7717/peerj.11882)
Supplement: Supplemental Information 8 [file peerj-09-11882-s008.docx]

**Table S2:**

**Pain groups diagnosis, medications and treatments**

| Participant | Diagnosis | Medications and/or treatments |
| --- | --- | --- |
| 1 | CRPS | Anti-inflammatories. Physiotherapy. |
| 2 | CRPS | Ketamine infusions, Amitriptyline, Pregabalin, Carbamazeprine, Calcitriol, Paracetamol, Codeine (when required), Lignocaine patches. Physiotherapy, meditation, hydrotherapy. |
| 3 | CRPS | Ketamine infusion, stellate ganglion nerve block, Lignocaine patches, Citalopram. TENS, acupuncture, physiotherapy. |
| 4 | CRPS | Intravenous Guanethidine blocks, Ketamine and Lignocaine infusions. Tramadol, Oxycodone-with-naloxone, Paracetamol-with-codeine, Alendronic acid. Physiotherapy, hydrotherapy, Graded Motor Imagery, Mirror Box therapy. |
| 5 | CRPS | Past: Pregabalin, Duloxetine. Current: Ibuprofen or Ibuprofen-and-codeine, Celecoxib, Diclofenac (oral and topical). PRN: Fentanyl patches. Physiotherapy, electro-interferential therapy, acupuncture, hot and cold therapy, ultrasound therapy. |
| 6 | CRPS | Nortriptyline, fish oil, Glucosamine. PRN: Paracetamol, Oxycodone, Ibuprofen. Physiotherapy. |
| 7 | CRPS | Amitriptyline. Physiotherapy, left/right discrimination exercises. |
| 8 | CRPS | Amitriptyline. Graded Motor Imagery, exercise, counselling. |
| 9 | CRPS | Gabapentin, Amitriptyline. PRN: Tilidine. Physiotherapy, mirror box therapy, paraffin wax therapy. |
| 10 | CRPS | Ketamine infusions, Pregabalin, Amitriptyline. Physiotherapy, mirror box therapy, pain education. |
| 11 | CRPS | Wearing day and night splints. Hand therapy. |
| 12 | CRPS | Past: Pregabalin. Current: Amitriptyline, Paracetamol-with-codeine, Paracetamol, Ibuprofen. PRN: Temazepam, Nitrazepam, Diazepam. Surgery, Occupational therapy, Physiotherapy, Hydrotherapy, Counselling, remedial massage. |
| 13 | CRPS | Paracetamol-with-codeine, Codeine. PRN: Pregabalin, Anti-inflammatories, Amitriptyline, Naloxone. Physiotherapy, hot and cold therapy, Occupational Therapy, Counselling, Relaxation techniques. |
| 14 | CRPS | Oxycodone-with-naloxone, Oxycodone, Diazepam, Paracetamol, Lignocaine patches. Counselling. |
| 15 | CRPS | Celecoxib, Duloxetine, Morphine sulphate, Nitrazepam. |
| 16 | CRPS | Gabapentin, Tapentadol, Amitriptyline. Pain specialist. |
| 17 | OA | Past: Paracetamol. Current: Glucosamine, fish oil. |
| 18 | OA | Past: Anti-inflammatories, Paracetamol, heat. Current: Occasional anti-inflammatories. |
| 19 | OA | Paracetamol, fish oil. Physiotherapy, acupuncture. |
| 20 | OA | Paracetamol |
| 21 | OA | Past: Diclofenac (topical). |
| 22 | OA | Denosumab injections. Natural remedies, massage, physiotherapy. |
| 23 | Osteoporosis | Osteoporosis medication (sic) |
| 24 | Carpal Tunnel Syndrome | Past: sound wave therapy. |
| 25 | Undiagnosed | Past: Physiotherapy, acupuncture, light therapy, massage, lymphatic drainage, hydrotherapy, comfrey cream, emu cream, aloe vera cream, vitamins. Current: vitamins, minerals. PRN: Paracetamol, Codeine. |
| 26 | Rheumatoid Arthritis | Past: Steroid injections, COX-2 inhibitors, immunosuppressants, Surgery to remove synovium, yoga, meditation, cold and hot patches, gloves, splints. Current medication: Leflunomide, Escitalopram. |
| 27 | OA | Past: anti-inflammatories. Current: Paracetamol, Codeine Phosphate Hemihydrate, Doxylamine succinate, Amitriptyline. |
| 28 | Juvenile Rheumatoid Arthritis, OA, Carpal Tunnel Syndrome | Paracetamol, aspirin. Heat, lotions, massage, meditation. |
| 29 | undiagnosed | Past: Rose hips capsules, aspirin. |
| 30 | OA | none |
| 31 | Undiagnosed | Past: cannabis, hand bandage. |
