# Supplementary material for: Where is my arm? Investigating the link between complex regional pain syndrome and poor localisation of the affected limb
Source: PeerJ. 2021 Aug 20;9:e11882. doi: 10.7717/peerj.11882 (PMC8381877; doi:10.7717/peerj.11882)
Supplement: Supplemental Information 9 [file peerj-09-11882-s009.docx]

**Table S3:**

**LMM1**

| AIC | | BIC | logLik | deviance | df. resid |  |
| --- | --- | --- | --- | --- | --- | --- |
| 23452.6 | | 23569.2 | -11706.3 | 23412.6 | 2500 |  |
|  | | | | | |  |
| **Scaled residuals:** | | | | | | |
|  | Min | | 1Q | Median | 3Q | Max |
|  | -4.2278 | | -0.545 | -0.0715 | 0.5425 | 5.3079 |
|  |  | | | | | |
| **Random effects:** | | | | | | |
|  | Groups | | Name | Variance | Std. Dev. |  |
|  | id | | (Intercept) | 86.1 | 9.279 |  |
|  | Residual | |  | 605.8 | 24.614 |  |
| Number of observations: 2520, Groups: id, 60 | | | | | |  |
|  | |  |  |  |  |  |
| **Fixed effects:** | | |  |  |  |  |
|  | | β | S.E. | df | t-value | p-value |
| (Intercept) | | 126.9754 | 3.2295 | 494.5359 | 39.317 | < 0.001 |
| Condition (Hidden) | | -114.527 | 3.8629 | 2460 | -29.648 | < 0.001 |
| Condition (Static) | | -114.542 | 3.8629 | 2460 | -29.652 | < 0.001 |
| Group (CRPS Pain) | | -2.2923 | 5.4161 | 494.5359 | -0.423 | 0.6723 |
| Group (non-CRPS pain) | | -1.042 | 5.5312 | 494.5359 | -0.188 | 0.8506 |
| Time | | -4.9846 | 0.6108 | 2460 | -8.161 | < 0.001 |
| Condition (Hid) x Group (CRPS) | | 12.0271 | 6.4783 | 2460 | 1.857 | 0.0635 |
| Condition (Stat) x Group (CRPS) | | 7.1892 | 6.4783 | 2460 | 1.11 | 0.2672 |
| Condition (Hid) x Group (non-CRPS Pain) | | 2.489 | 6.616 | 2460 | 0.376 | 0.7068 |
| Condition (Stat) x Group (non-CRPS Pain) | | 0.7419 | 6.616 | 2460 | 0.112 | 0.9107 |
| Condition (Hid) x Time | | 6.9175 | 0.8638 | 2460 | 8.008 | < 0.001 |
| Condition (Stat) x Time | | 6.3596 | 0.8638 | 2460 | 7.363 | < 0.001 |
| Group (CRPS) x Time | | -0.8703 | 1.0243 | 2460 | -0.85 | 0.3956 |
| Group (non-CRPS) x Time | | -4.5237 | 1.0461 | 2460 | -4.324 | < 0.001 |
| Condition (Hid) x Group (CRPS) x Time | | 0.0122 | 1.4486 | 2460 | 0.008 | 0.9933 |
| Condition (Stat) x Group (CRPS) x Time | | 1.0991 | 1.4486 | 2460 | 0.759 | 0.4481 |
| Condition (Hid) x Group (non-CRPS Pain) x Time | | 3.2575 | 1.4794 | 2460 | 2.202 | < 0.05 |
| Condition (Stat) x Group (non-CRPS Pain) x Time | | 5.8975 | 1.4794 | 2460 | 3.987 | < 0.001 |
