# Supplementary material for: Where is my arm? Investigating the link between complex regional pain syndrome and poor localisation of the affected limb
Source: PeerJ. 2021 Aug 20;9:e11882. doi: 10.7717/peerj.11882 (PMC8381877; doi:10.7717/peerj.11882)
Supplement: Supplemental Information 10 [file peerj-09-11882-s010.docx]

**Table S4:**

**LMM2**

|  | AIC | BIC | logLik | deviance | df. resid |
| --- | --- | --- | --- | --- | --- |
|  | 12206.9 | 12465.5 | -6053.4 | 12106.9 | 1252 |
|  |  |  |  |  |  |
|  | **Scaled residuals:** | | | | |
|  | Min | 1Q | Median | 3Q | Max |
|  | -3.7428 | -0.5159 | -0.0902 | 0.5134 | 4.1434 |
|  |  |  |  |  |  |
|  | **Random effects:** | | | |  |
|  | Groups | Name | Variance | Std.Dev. |  |
|  | id | (Intercept) | 70.52 | 8.397 |  |
|  | Residual |  | 613.29 | 24.765 |  |
|  | Number of observations: 1302, Groups: id, 31 | | | |  |
|  |  |  |  |  |  |
| **Fixed effects:** |  |  |  |  |  |
|  | β | S.E. | df | t-value | p-value |
| (Intercept) | -7.9E+00 | 1.73E+01 | 1.05E+02 | -0.455 | 0.65015 |
| Group (non-CRPS Pain vs. CRPS Pain) | 3.65E+01 | 4.54E+01 | 1.05E+02 | 0.804 | 0.42323 |
| Hand (Affected vs. Unaffected) | 2.51E+01 | 1.82E+01 | 1.27E+03 | 1.378 | 0.16847 |
| Condition (DHT) | 1.49E+02 | 1.82E+01 | 1.27E+03 | 8.21 | <0.001 |
| Condition (Static) | -5.75E+00 | 1.82E+01 | 1.27E+03 | -0.316 | 0.7521 |
| Age | 1.31E-01 | 4.01E-01 | 1.05E+02 | 0.327 | 0.74426 |
| Anxiety | 4.53E-01 | 6.58E-01 | 1.05E+02 | 0.689 | 0.49225 |
| Group (non-CRPS) x Hand (affected) | -2.87E+01 | 4.78E+01 | 1.27E+03 | -0.601 | 0.54816 |
| Group (non-CRPS) x Condition (DHT) | -1.15E+02 | 4.78E+01 | 1.27E+03 | -2.397 | <0.05 |
| Group (non-CRPS) x Condition (Static) | 9.18E+00 | 4.78E+01 | 1.27E+03 | 0.192 | 0.84764 |
| Hand (Affected) x Condition (DHT) | -5.45E+01 | 2.57E+01 | 1.27E+03 | -2.118 | <0.05 |
| Hand (Affected) x Condition (Static) | 5.07E+00 | 2.57E+01 | 1.27E+03 | 0.197 | 0.84367 |
| Group (non-CRPS) x Age | -2.81E-01 | 7.99E-01 | 1.05E+02 | -0.352 | 0.72525 |
| Hand (Affected) x Age | -3.72E-01 | 4.22E-01 | 1.27E+03 | -0.882 | 0.37812 |
| Condition (DHT) x Age | -1.82E+00 | 4.22E-01 | 1.27E+03 | -4.32 | <0.001 |
| Condition (Static) x Age | 8.06E-01 | 4.22E-01 | 1.27E+03 | 1.912 | 0.05613 |
| Group (non-CRPS) x Anxiety | -2.12E+00 | 2.18E+00 | 1.05E+02 | -0.972 | 0.33343 |
| Hand (Affected) x Anxiety | -4.99E-01 | 6.92E-01 | 1.27E+03 | -0.721 | 0.47088 |
| Condition (DHT) x Anxiety | -3.03E-01 | 6.92E-01 | 1.27E+03 | -0.437 | 0.66203 |
| Condition (Static) x Anxiety | 1.64E-01 | 6.92E-01 | 1.27E+03 | 0.237 | 0.81256 |
| Age x Anxiety | 1.79E-02 | 1.52E-02 | 1.05E+02 | 1.18 | 0.24077 |
| Group (non-CRPS) x Hand (Affected) x Condition (DHT) | -1.65E+01 | 6.75E+01 | 1.27E+03 | -0.245 | 0.80679 |
| Group (non-CRPS) x Hand (Affected) x Condition (Static) | -5.58E+00 | 6.75E+01 | 1.27E+03 | -0.083 | 0.93414 |
| Group (non-CRPS) x Hand (Affected) x Age | 4.43E-01 | 8.41E-01 | 1.27E+03 | 0.527 | 0.5985 |
| Group (non-CRPS) x Condition (DHT) x Age | 2.27E+00 | 8.41E-01 | 1.27E+03 | 2.7 | <0.005 |
| Group (non-CRPS) x Condition (Static) x Age | -7.71E-01 | 8.41E-01 | 1.27E+03 | -0.917 | 0.35924 |
| Hand (Affected) x Condition (DHT) x Age | 1.41E+00 | 5.97E-01 | 1.27E+03 | 2.361 | <0.05 |
| Hand (Affected) x Condition (Static) x Age | -4.04E-01 | 5.97E-01 | 1.27E+03 | -0.676 | 0.49893 |
| Group (non-CRPS) x Hand (Affected) x Anxiety | 7.02E-01 | 2.29E+00 | 1.27E+03 | 0.306 | 0.75958 |
| Group (non-CRPS) x Condition (DHT) x Anxiety | 4.77E+00 | 2.29E+00 | 1.27E+03 | 2.079 | <0.05 |
| Group (non-CRPS) x Condition (Static) x Anxiety | -5.95E-02 | 2.29E+00 | 1.27E+03 | -0.026 | 0.97932 |
| Hand (Affected) x Condition (DHT) x Anxiety | 1.31E+00 | 9.79E-01 | 1.27E+03 | 1.338 | 0.18105 |
| Hand (Affected) x Condition (Static) x Anxiety | -2.70E-01 | 9.79E-01 | 1.27E+03 | -0.275 | 0.78298 |
| Group (non-CRPS) x Age x Anxiety | 8.48E-03 | 3.82E-02 | 1.05E+02 | 0.222 | 0.82486 |
| Hand (Affected) x Age x Anxiety | -7.44E-03 | 1.60E-02 | 1.27E+03 | -0.465 | 0.64186 |
| Condition (DHT) x Age x Anxiety | -7.50E-03 | 1.60E-02 | 1.27E+03 | -0.469 | 0.63921 |
| Condition (Static) x Age x Anxiety | -3.11E-02 | 1.60E-02 | 1.27E+03 | -1.946 | 0.05192 |
| Group (non-CRPS) x Hand (Affected) x Condition (DHT) x Age | -2.86E-01 | 1.19E+00 | 1.27E+03 | -0.241 | 0.80987 |
| Group (non-CRPS) x Hand (Affected) x Condition (Static) x Age | 4.46E-01 | 1.19E+00 | 1.27E+03 | 0.375 | 0.70743 |
| Group (non-CRPS) x Hand (Affected) x Condition (DHT) x Anxiety | 1.460e+00 | 3.24E+00 | 1.27E+03 | 0.45 | 0.65264 |
| Group (non-CRPS) x Hand (Affected) x Condition (Static) x Anxiety | 1.13E+00 | 3.24E+00 | 1.27E+03 | 0.349 | 0.72745 |
| Group (non-CRPS) x Hand (Affected) x Age x Anxiety | 2.98E-03 | 4.02E-02 | 1.27E+03 | 0.074 | 0.9409 |
| Group (non-CRPS) Condition (DHT) x Age x Anxiety | -6.29E-02 | 4.02E-02 | 1.27E+03 | -1.564 | 0.11807 |
| Group (non-CRPS) x Condition (Static) x Age x Anxiety | 3.06E-02 | 4.02E-02 | 1.27E+03 | 0.76 | 0.44736 |
| Hand (Affected) x Condition (DHT) x Age x Anxiety | -9.32E-03 | 2.26E-02 | 1.27E+03 | -0.412 | 0.68031 |
| Hand (Affected) x Condition (Static) x Age x Anxiety | 2.36E-02 | 2.26E-02 | 1.27E+03 | 1.043 | 0.2973 |
| Group (non-CRPS) x Hand (Affected) c Condition (DHT) x Age x Anxiety | 3.022e-02 | 5.69E-02 | 1.27E+03 | -0.531 | 0.59528 |
| Group (non-CRPS) x Hand (Affected) x Condition (Static) x Age x Anxiety | -4.136e-02 | 5.69E-02 | 1.27E+03 | -0.727 | 0.46727 |
